# To optimize gas flaring in Kirkuk refinery in various seasons via artificial intelligence techniques

**DOI:** 10.1038/s41598-023-40724-2

**Published:** 2023-08-17

**Authors:** A. Zoeir, J. Qajar, Y. Kazemzadeh, E. Khodapanah, A. Rastkar

**Affiliations:** 1 Department of Petroleum Engineering, School of Chemical and Petroleum Engineering, Shiraz University, Shiraz, Iran; 2 Department of Petroleum Engineering, Persian Gulf University, Bushehr, Iran; 3 Faculty of Petroleum and Natural Gas Engineering, Sahand University of Technology, Tabriz, Iran

**Keywords:** Chemical engineering, Energy, Environmental chemistry

## Abstract

Unavoidable flaring in downstream oil industry causes pollutant emission in large amounts which is potentially harmful to nearby cities or farms. Hence one must manage exhaust toxic gases to raise enough in atmosphere or redirect from such places. Since Kirkuk refinery in north Iraq is next-door to agricultural farms on west yet to residential areas on east optimizing its layout for flare stacks is something acute. In this work we wrote codes in MATLAB software to simulate incomplete rather than complete oxidation as well as pollutant generation reactions. Then we made use of FLEUENT software to simulate pollutant propagation in Kirkuk oil purifier complex yet also farther to city as well as farms with respect to seasonal air currents on lowest troposphere layer. Finally, we set neural network approach to train on simulation data thereafter to unify outcomes to turn into a fast technique for layout optimization. Results show that optimization process efficiency relies on air current velocities as well as its direction. At intermediate air flow rates optimum layout includes only a selective portion of existent flare stacks. Outcomes also illustrate that heuristic techniques that have stronger local search such as particle swarm or artificial immune system can improve flare layout in seasons with intermediate air currents here summer plus early months in autumn while approaches with weak local search like Monte Carlo are more appropriate in winter for which we have no or low air flows in Kirkuk governorate.

## Introduction

In 2010 ten countries were at fault for 72% of total sour gas flares internationally. Top four countries were Russia (27%) then Nigeria (11%) thereafter Iran (8%) yet at last Iraq (7%). Data from Satellites show that overall gas flaring fell to about 80% in recent few years. The most significant reductions in terms of volume were made in Russia (up to 40%) then Nigeria (up to 29%). One can expect Iraq to run up the list in next few years. Kirkuk refinery as largest oil complex in northern Iraq emits more than 70 million ${\mathrm{ft}}^{3}/\mathrm{D}$ air contaminant gases to the atmosphere. Since this complex is next-door to agricultural farms on west as well as residential areas in Kirkuk city on east optimizing its flare system layout is deeply acute. Accessibility issue often forces us to place oil refineries or petrochemical complexes close to the urban districts which causes several environmental difficulties like diseases in social communities. Literature shows that investigators had various tries to optimize flare system layout as well as to reduce pollutant exhaust gases. In this way for instance Abdulkareem^1^ made use of Gaussian functions to simulate air pollutants dispersion in an industrial zone full of flares via Visual Basic programming language. It was shown that air pollutant emission is firmly dependent on flue gas flow rate rather than airflow velocity as well as distance from flare stacks^1^. Alameddine et al.^2^ made use of an industrial complex air pollutant dispersion model to estimate sulfur oxide concentrations in a power distillation plant in an industrial area. It was shown that whenever flare stacks have different heights or in situations with low or no air currents more contaminants emit in near environment^2^. Yassin et al.^3^ set tunnel apparatus to simulate pollutant dispersion in a residential area in Japan under various air flow conditions. Outcomes show that under low or no air flow condition pollutant diffusion was high in comparison to situations with high air currents^3^. Lee^4^ developed several codes in FORTRAN language to simulate pollutants emission via diffusion on an uneven surface under turbulent condition. Outcomes display that local pollutant mass fractions are highly sensitive to location of pollution source as well as existent structures^4^. Nazirdoust et al.^5^ made use of FLUENT software to simulate pollutant dispersion in residential areas with tall structures. Results show that the windy face of constructions is has less pollutant mass fractions yet structure heights are effective on pollutant propagation^5^. Perez-Roa et al.^6^ set artificial neural network to train on several pollutant measurement data plus simulation results thereafter made use of this intelligent network to trace $\mathrm{CO}$ concentrations in capital city of Chile. It was shown that simulation results are in agreement with experimental data in almost all days except Saturday to Sunday for which uncertainties were high^6^. Kahforoshan et al.^7^ developed codes in MATLAB software to study to what extent factors like flare height or air conditions can affect pollutant emission in an industrial area in Africa. It was shown that forecasts from simulations were in nice agreement with experimental information on $\mathrm{NO}$ rather than ${\mathrm{SO}}_{2}$ mass fractions^7^. Edokpa et al.^8^ did several sensitivity analyses on parameters like air flow velocity rather than direction in Niger delta of Nigeria via employing a commercial simulator. It was shown that higher air flow velocity as well as lower sour gas flow rate causes higher pollutant concentrations near the flare rather than farther locations^8^. Alkaim et al.^9^ set data mining technique down with optimization algorithms which relies on multivariate adaptive regression technique to achieve optimal gas flaring rate with respect to costs. It was shown that this approach was very fast yet powerful since it applies integration function^9^. Fawole et al.^10^ did an study to discover to what extent stack height or gas composition influences pollutant emission in the Niger delta. Outcomes illustrate that the density of fuel has reverse relation with zero level pollutants concentration while authors propose using a taller flare stack in lieu of a shorter one^10^. Zoeir et al.^11^ wrote several codes in MATLAB software to model complete rather than incomplete oxidation reaction then to minimize pollutants in flue gases. Outcomes illustrate that there is specific value for excess air for which mass fractions of air contaminants namely ${\mathrm{NO}}_{\mathrm{x}}$ plus ${\mathrm{SO}}_{\mathrm{x}}$ cuts down^11^. Baroutian et al.^12^ made use of Gaussian plume model to forecast pollutant dispersion from cement plant to residential areas of Kerman city in southeastern Iran thereafter did a comparison with real data that came from measurements. Since predictions via Gaussian plume model were in nice agreement with data it was a proof that this approach is rational elsewhere under low air currents^12^. Abiye et al.^13^ set sensors to measure air pollutants in an iron recycling factory that uses fossil fuels as main energy source thereafter made estimations via plume dispersion simulation in other places for which sensors did not exist. Sensitivity analyses to air temperature in addition to net radiation illustrate that that locations with intermediate distance from the scrap-iron recycling factory were most prone to the impacts of the gaseous releases from the factory’s operations^13^. Ismail et al.^14^ wrote several computer programs to simulate incomplete rather than complete oxidations in addition to pollutant generation reactions for ${\mathrm{SO}}_{2}$ plus ${\mathrm{NO}}_{\mathrm{x}}$ as well as ${\mathrm{CO}}_{\mathrm{x}}$. it was shown that the quantity of these chemical species relies on percentage of excess air or deficiency of stoichiometric air yet natural gas composition together with impurity contents^14^.

Main novelties of this study in comparison to other works in the literature are that here we employ heuristic optimization techniques to detect monthly optimal flare layout in Kirkuk governorate. Here we concern not only the air currents in lowest troposphere layer yet also simulate pollutant migration through an upper layer with powerful air currents that do not sense earth’s features. In addition to that we develop an intelligent technique namely neural network to rapidly forecast optimum layout for any specific daily air current without requiring to perform computational dynamics. Moreover, we do several sensitivity analyses on factors like air flow air flow characteristics or sour gas flow rates to investigate to what extent each optimization algorithm is efficient.

## Methodology

**Figure 1:**
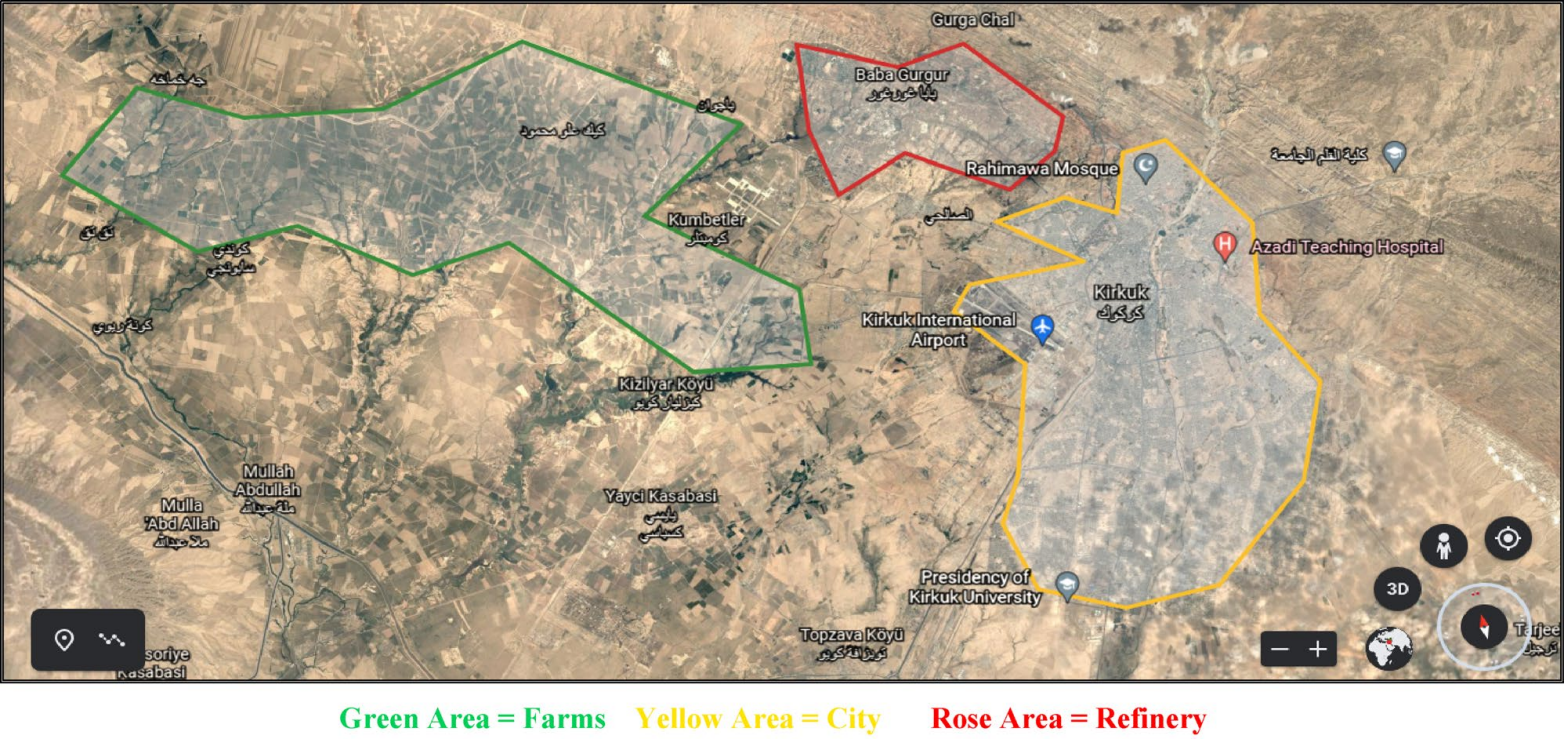
Layout for Kirkuk refinery taken from Google earth.

In this section we firstly explain our industrial case study in northern Iraq with further details on stack locations in addition to average sour gas composition. Then we report information on local seasonal rather than permanent air currents that are taken from NASA online site (Fig. 1). To coincidentally minimize pollutant transport to residential areas as well as agricultural farms we employ heuristic optimization techniques. Such approaches search for optimum specifics like intake flow rates or excess air to each flare stack yet which ones to turn off or on with respect to seasonal air currents. In the next step neural network approach was chosen to unify information on optimal flare layout that comes from computations for each month. In all our calculations we use Fluent software to perform computational dynamics thereafter to employ its results on pollutant mass fractions in two target areas as test function. Finally, we employ artificial intelligence to perform several sensitivity analyses on factors like air current velocity or direction in addition to intake sour gas flow rate to investigate to what extent is the process efficiency.

To speak on data, one must firstly explain the case study for which we want to optimize flare stack layout. Kirkuk refinery meets residential areas on its east to south east as well as farms on west. Both areas suffer from air pollutants in high concentration due to their proximity if air currents in troposphere cannot effectively carry contaminants away. Pollutant emission with high mass fractions in Kirkuk city causes several diseases such as stroke or cancer in society thus directly affects lives. This is while high pollutant concentrations in plantations or farms indirectly influence fauna with taint on crops. Our further data is on locations of flare stacks that are currently set in place in Kirkuk refinery limits. In order to represent locations, we use global positioning system that employs geographical factors namely latitudinal values for distances from equator line in addition to longitudinal values for distances from prime meridian line as well as altitudinal amounts for height from free sea level. Here mainly due to economical purposes we perform optimization computations on existent flares via changing they’re on or off status rather than excess air to detect layouts that minimize pollutant mass fraction in two target areas. Certainly, one can even consider potential to place new stacks in refinery limits which surely increases capital investments. Further details on flare positions plus their current status is shown in Table 1.

**Table 1:** Information on existent flare stacks in Kirkuk refinery.

Location			Status
Latitude	Longitude	Attitude (ft)	
35°31′36′′N	44°20′45′′E	1096	On
35°32′04′′N	44°20′12′′E	1119	On
35°32′02′′N	44°20′14′′E	1090	On
35°31′50′′N	44°20′21′′E	1086	On
35°31′40′′N	44°20′32′′E	1090	On
35°31′38′′N	44°20′34′′E	1096	Off
35°32′21′′N	44°19′43′′E	1106	Off
35°32′23′′N	44°19′42′′E	1116	Off
35°31′23′′N	44°20′40′′E	1080	On
35°31′25′′N	44°20′55′′E	1077	Off
35°31′56′′N	44°20′19′′E	1102	Off

To discuss seasonal local air currents in Kirkuk governorate we use data from NASA online site that not only includes surface air currents yet also explains air flow velocities in higher troposphere layers. Information tells us that during early spring in lowest troposphere layer we see no or extremely low air currents while after two- or three-months seasonal air flows start to rush from northwest. In summer we have powerful flows with high velocities from north to south for 2 months after that air currents weaken for 1 month. In autumn delicate air currents direct from northeast to southwest. Finally in winter air currents slow down to reach trivial velocities prior to spring. This is while in upper troposphere layers air flows are not so much relevant to seasons. In contrast there we have extremely powerful air currents that are always from west to east. If one wants to explain why air flows near the surface are so uncertain the answer lies under local topological features such as mountain or plateaus.

**Figure 2:**
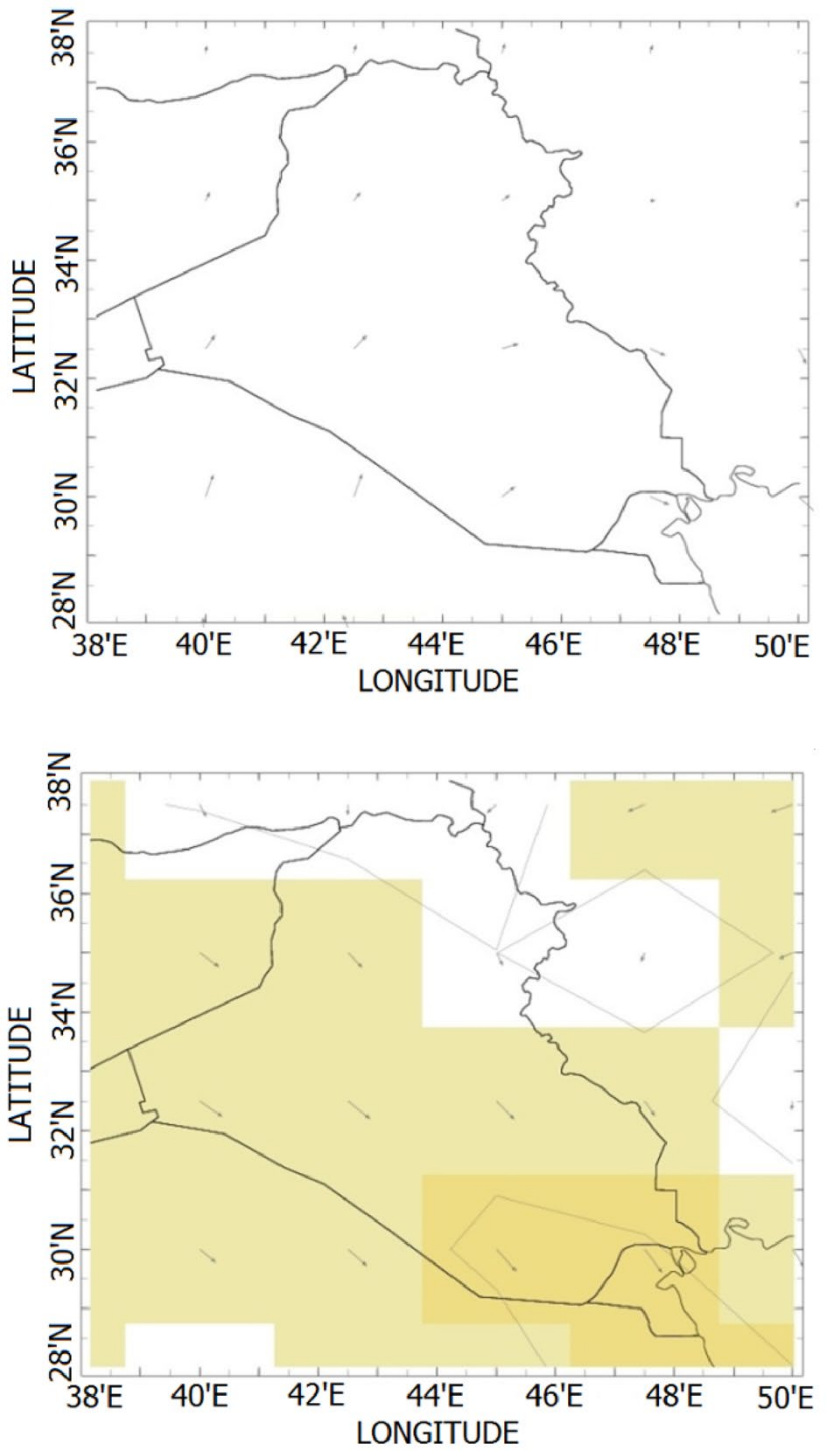
Local air currents in Kirkuk Governorate for April (upper) & June (lower).

Since provincial hills or valleys are always there, we can expect local air currents to do not undergo significant yearly changes. Anyway, daily currents may not exactly follow this algorithm due to impermanent reasons such as unusual temperature profiles or other factors. Here we introduce two exemplar air current maps for April rather than June as shown in Fig. 2. One can look at Table 2 for further details on air current velocities.

**Table 2:** Monthly air current information in Kirkuk Governorate.

		Troposphere layer	
		Lower (MPH)	Upper (MPH)
Month	Direction	Velocity	
Jan	No	0	12
Feb	No	0	13
Mar	No	0	14
Apr	Western	2	16
May	Northwestern	3	17
Jun	Northwestern	6	19
Jul	Northwestern	4	18
Aug	Northwestern	3	17
Sep	Northern	3	14
Oct	Northern	2	13
Nov	Northeastern	2	14
Dec	No	0	13

Since Kirkuk complex is the largest oil clarifier unit in northern Iraq it vents large amounts of sour gas each year. Corrosive components in streamline prior to oxidation reaction are ${\mathrm{CO}}_{2}$ rather than ${\mathrm{H}}_{2}\mathrm{S}$ that in coexistence with ${\mathrm{H}}_{2}\mathrm{O}$ cause extreme taints. However, three-phase separators that are in line prior to flare stacks remove aqueous phase as free water stream.

Further details on sour gas compositions are shown in Table 3.

**Table 3:** Intake sour gas composition in Kirkuk refinery.

Fractions (Mole %)	Sour gas (%)
Component	
Carbon dioxide	3.5
Nitrogen	0.5
Hydrogen sulphide	2.8
Methane	90.6
Ethane	0.8
Propane	0.3
Butane	0.2
Pentane	0.3
Hexane	0.2
Heptane	0.0
Impurities	0.8

To compute the amount of greenhouse gases as well as air pollutants that emit from flare stacks, we simulate fuel oxidation reaction to attain flue gas composition. Each hydrocarbon component within inlet sour gaseous phase participates in an exothermic reaction which in the presence of enough excess air will go through complete oxidation that yields only ${\mathrm{CO}}_{2}$ in addition to ${\mathrm{H}}_{2}\mathrm{O}$. General formulation for complete oxidation is:

$${\mathrm{C}}_{\mathrm{x}}{\mathrm{H}}_{\mathrm{y}}+{\mathrm{zO}}_{2}+3.77{\mathrm{zN}}_{2}+{\mathrm{iCO}}_{2}+{\mathrm{jN}}_{2}\to$$

1 $${\mathrm{xCO}}_{2}+0.5{\mathrm{yH}}_{2}\mathrm{O}+3.77{\mathrm{zN}}_{2}+{\mathrm{iCO}}_{2}+{\mathrm{jN}}_{2}$$

Conversely in situations with less than enough oxidant hydrocarbon components go through incomplete oxidation that produces ${\mathrm{H}}_{2}$ as well as $\mathrm{CO}$ in addition to other routine products. General form for incomplete oxidation is:

$${\mathrm{C}}_{\mathrm{x}}{\mathrm{H}}_{\mathrm{y}}+{\mathrm{zO}}_{2}+3.77{\mathrm{zN}}_{2}+{\mathrm{iCO}}_{2}+{\mathrm{jN}}_{2}\to$$

2 $${\mathrm{wCO}}_{2}+\mathrm{vCO}+{\mathrm{pH}}_{2}\mathrm{O}+{\mathrm{qH}}_{2}+3.77{\mathrm{zN}}_{2}+{\mathrm{iCO}}_{2}+{\mathrm{jN}}_{2}$$

One must calculate enthalpy changes relevant to each fuel component from thermodynamics that concerns difference in free energy that determines whether reaction is exothermic or not. Conversely reaction rates for reactants come from kinetics via considering especial orders yet constants for each fuel component within its cremation. Enthalpy values for all hydrocarbon components are negative which exhibits exothermic oxidations whereas also reaction rates show their instant conversion. Conversely ${\mathrm{N}}_{2}$, ${\mathrm{O}}_{2}$ as well as ${\mathrm{H}}_{2}\mathrm{S}$ oxidation to ${\mathrm{NO}}_{\mathrm{x}}$ or ${\mathrm{SO}}_{\mathrm{x}}$ disobey the above rules. Nitrogen oxides which are also known as ${\mathrm{NO}}_{\mathrm{x}}$ emit from flares in form of $\mathrm{NO}$ (about 90–95%) yet much less in form of ${\mathrm{NO}}_{2}$ (about 5–10%). Other nitrogen oxides like nitrous oxide appear only in negligible amounts in flare stacks. Two formation mechanisms namely prompt in front of thermal contribute in the formation of nitric oxide from the oxidant resource. Prompt mechanism presents that firstly some available radicals react with nitrogen molecule to form $\mathrm{HCN}$, $\mathrm{HN}$ as well as $\mathrm{CN}$ molecules thereafter such products easily oxidize to nitric oxide. Conversely thermal mechanism presents that some high energy molecules dissociate oxygen molecule to form oxygen radial then these radical attacks nitrogen molecule to form nitric oxide. Nevertheless, to what extent each mechanism participates in ${\mathrm{N}}_{2}$ oxidation, we adjust $\mathrm{NO}$ formation equation in our code in the form of:

3 $${\mathrm{xO}}_{2}+{\mathrm{xN}}_{2}\to 2\mathrm{xNO}$$

While slow spontaneous conversion of nitric oxide to nitrogen dioxide takes place in small amounts according to exothermic equation of form:

4 $$\mathrm{xNO}+{\mathrm{xO}}_{2}\to 2{\mathrm{xNO}}_{2}$$

It is noticeable that the overall ${\mathrm{NO}}_{\mathrm{x}}$ formation is definitely endothermic so when adiabatic flame temperature is high nitrogen oxidation accelerates leading to more ${\mathrm{NO}}_{\mathrm{x}}$ emission.

Sour flares intake ${\mathrm{H}}_{2}\mathrm{S}$ that oxidizes into sulphur dioxide yet trioxide. If oxygen is present in high amounts dominant product is ${\mathrm{SO}}_{3}$ whereas less oxygen forms much ${\mathrm{SO}}_{2}$. Both reactions are exothermic. Sulfur dioxide participates in ${\mathrm{H}}_{2}{\mathrm{SO}}_{3}$ formation in acidic rains while ${\mathrm{SO}}_{3}$ conspire in ${\mathrm{H}}_{2}{\mathrm{SO}}_{4}$ formation thus illustrates corrosive characteristics. Oxidation reactions are similar to each other with this difference that ${\mathrm{SO}}_{3}$ formation consumes more oxygen. We consider them as:

$$2{\mathrm{xH}}_{2}\mathrm{S}+3{\mathrm{xO}}_{2}\to 2{\mathrm{xSO}}_{2}+{2\mathrm{xH}}_{2}\mathrm{O}$$

5 $${\mathrm{xH}}_{2}\mathrm{S}+2{\mathrm{xO}}_{2}\to {\mathrm{xSO}}_{3}+{\mathrm{xH}}_{2}\mathrm{O}$$

**Figure 3:**
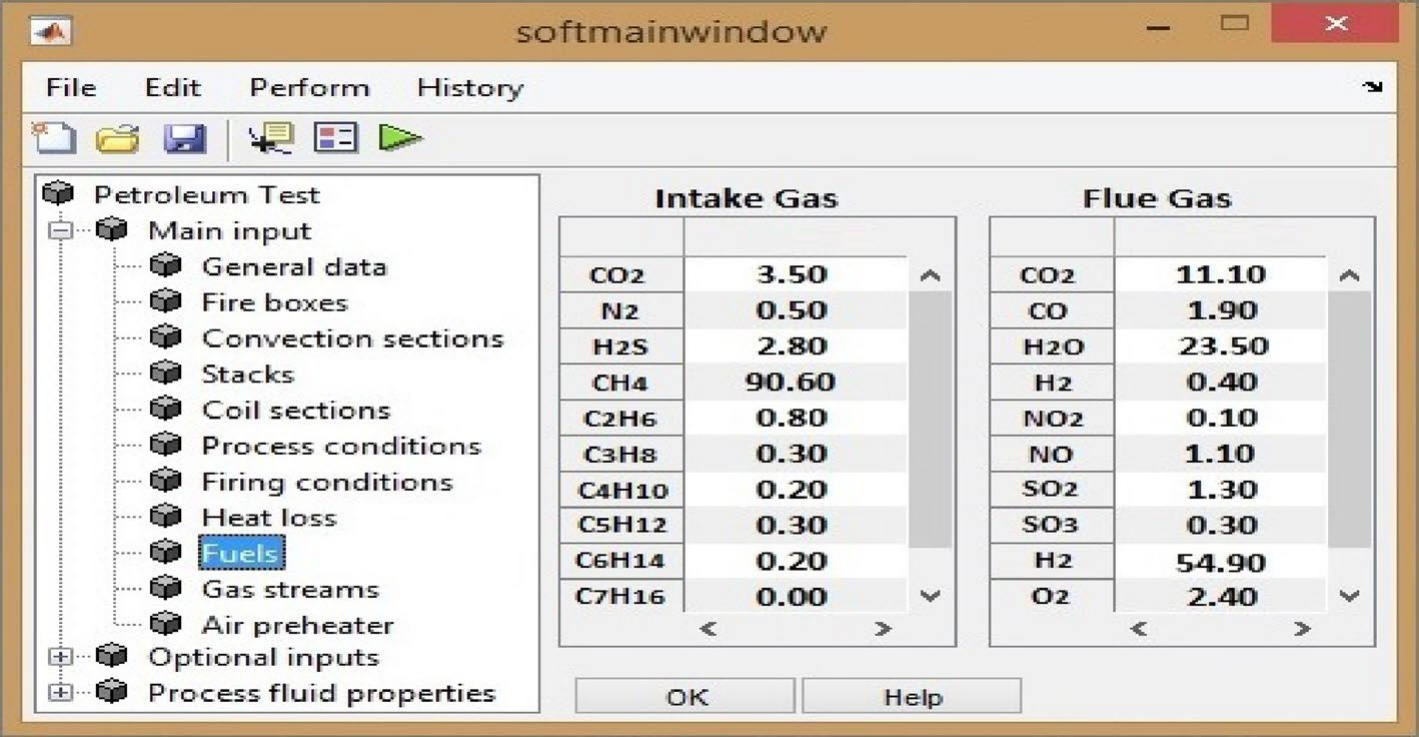
Our in-house program that calculates flue gas compositions.

In order to perform reaction calculations, one has two alternatives. First way is to write an independent program or apply a convenient software for such purpose. Next option is to do oxidation calculations in the same software that performs computational dynamics. Here we chose to develop an in-house MATLAB program that gives us flame temperature as well as flue gas composition with respect to excess air as shown in Fig. 3.

**Figure 4:**
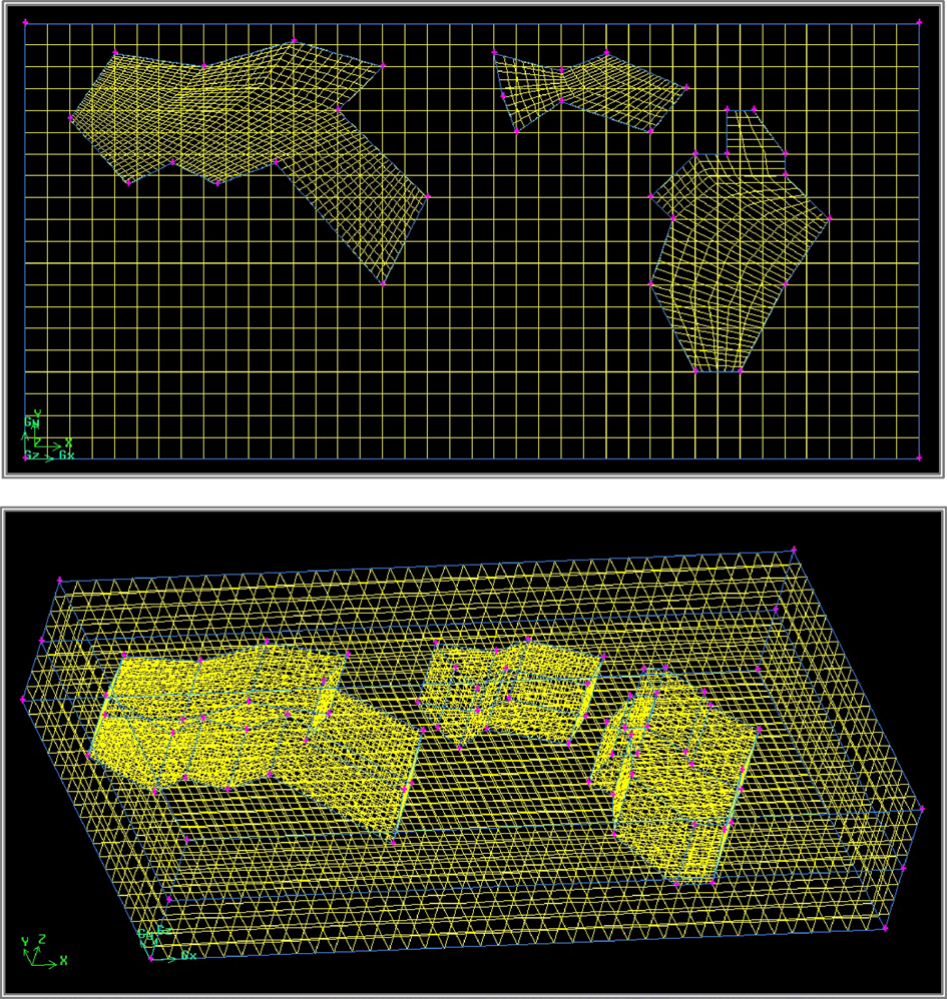
Our prototype in GAMBIT for computational dynamics top view (upper) 3D view (lower).

In this step we know flame temperature for each flare in addition to information on flue gas composition for all existent stacks with known locations. Now is the time to simulate pollutant dispersion from Kirkuk refinery to its environs namely adjacent city on east as well as farms on west. To do this one must employ computational dynamics that unites mass with energy conservation equations to predict flow specifications in systems. For this purpose, we use GAMBIT software that designs structures in accompaniment with Fluent software that employs dominant laws to do computational dynamics. In this section we develop a three-dimensional prototype that simulates to what extent air contaminant can reach residential area or agricultural farms. Main reason to use spatial geometry rather than two dimensional areal models is that we want to know how each pollutant component lowers its height when cools down in environment. In addition to that since here we have several flare stacks at once one cannot use vertical cross section geometry in this study. Therefore, we prepare three dimensional prototypes that considers not only refinery limits yet urban regions on Kirkuk city as well as next door agricultural farms as shown in Fig. 4. Sensitive regions such as refinery itself or target areas require more attention yet accuracy thus finer mesh while other regions do not. One can detect other farms in south western Kirkuk city much farther from refinery that we exclude from our calculations. Our reason is that such farms are in places that air pollutants from Kirkuk flares cannot provide high concentrations.

All our simulations in this case study undergo steady state condition since times to turn on or off flares are extremely small in comparison to operation times. By applying computational dynamics, we appraise average concentration for each contaminant component namely ${\mathrm{NO}}_{\mathrm{x}}$ ${\mathrm{SO}}_{\mathrm{x}}$ as well as ${\mathrm{CO}}_{2}$ or $\mathrm{CO}$ in our target areas. Here we consider two different layers that lie on each other. Lower layer expresses lowest air film in troposphere that covers surface plus its features like mountains or valleys. Upper layer expresses the high velocity air layer in troposphere that does not sense any earth’s features. To detect an optimal layout for flares that are present in our case study we start from an initial state that is set stochastically via using mathematical random functions. In this state certain flares that collect values larger than half of unity are alive while others are set off. Next step is to do computational dynamics for such a layout to calculate average air pollutant mass fractions in two target areas namely city on east as well as farms on west. We perform these calculations in FLUENT software which intakes flue gas characteristics plus air flow conditions then uses mass in cooperation with energy conservation equations to determine pollutant emission in target areas. These values are evaluation functions that specify whether a certain layout is superior to other patterns or not. Next step in optimization process is to search for another more proper layout. To do this one must undergo local or overall search procedure. Here we define local search as situation in which one of current alive flares sets off or a next-door offline flare turns on. Conversely, we define overall search to reuse stochastic mathematical functions for another guess on status of all flares. If our new layout nevertheless comes from a local or an overall search step displays lower average pollutant mass fraction in target areas, we replace the previous layout with new one. Otherwise, we keep previous one as our temporary optimal solution. We made use of four metaheuristic algorithms with unalike local or overall search strengths to detect optimal flare layout as is shown in Table 4.

**Table 4:** Artificial intelligence techniques.

Search algorithms
Artificial intelligence techniques
Monte carlo
Genetic algorithm
Particle swarm optimization
Artificial immune system
Unifying information
Neural network

Monte Carlo technique relies on fortuitous sampling from solution space in overall scale which is here statuses of flares. Two crucial issues that appear in applying this technique are weak local search as well as requirement for massive random tries to detect an optimal solution. This is while genetic algorithm starts with an initial random try to set flare statuses thereafter evaluates target functions to select top candidates among all initial tries. Process continues with changing statuses for one next-door flare stack from layout that comes from previous step. Genetic algorithm improves layout with advancing local search rather than overall that may result premature solutions. Particle swarm optimization randomly places set values for flare statuses in each layout to discover most valuable layout via evaluating pollutant concentrations. This metaheuristic technique defines a velocity vector for each flare stack with respect to its status in interim optimal layout that updates after each try.

In last step of our computations, we made use of neural network approach to unify monthly information that came from search in our case study for optimal layout. This approach is a pure mathematical therefore fast technique which exactly relates intake factors to outlet results. Since neural network neglects all the previous conceptual laws it is most useful in solution spaces with unknown governing equations. Here we use seasonal air direction rather than average monthly velocities as two most significant inputs. Beside them we also import excess air for each flare that computes flame temperature together with statuses of flare stacks in each optimal layout. One can expect more accurate outcomes with less intake parameters yet respectively more train or test instances. Here we employ 80% of exemplar data taken from monthly searches as train data while leaving 20% for test. Finally, we employ this intelligent network to perform sensitivity analyses on mean air flow velocity rather than its direction in addition to total intake volumetric flow rate.

## Discussing outcomes

In this section firstly we verify our outcomes against routine pattern for which all flares are inline. In order to do this, we draw seasonal pollutant concentrations in two target regions namely city on east as well as farms on west. Thereafter we illustrate two exemplar optimal layouts for two specific circumstances for local seasonal air currents. After that we explore to what extent each search algorithm optimizes alive flare pattern as iterations go on. Farther in this section we perform several sensitivity analyses over air current velocity rather than direction as well as intake sour gas flow rate via applying neural network approach.

**Figure 5:**
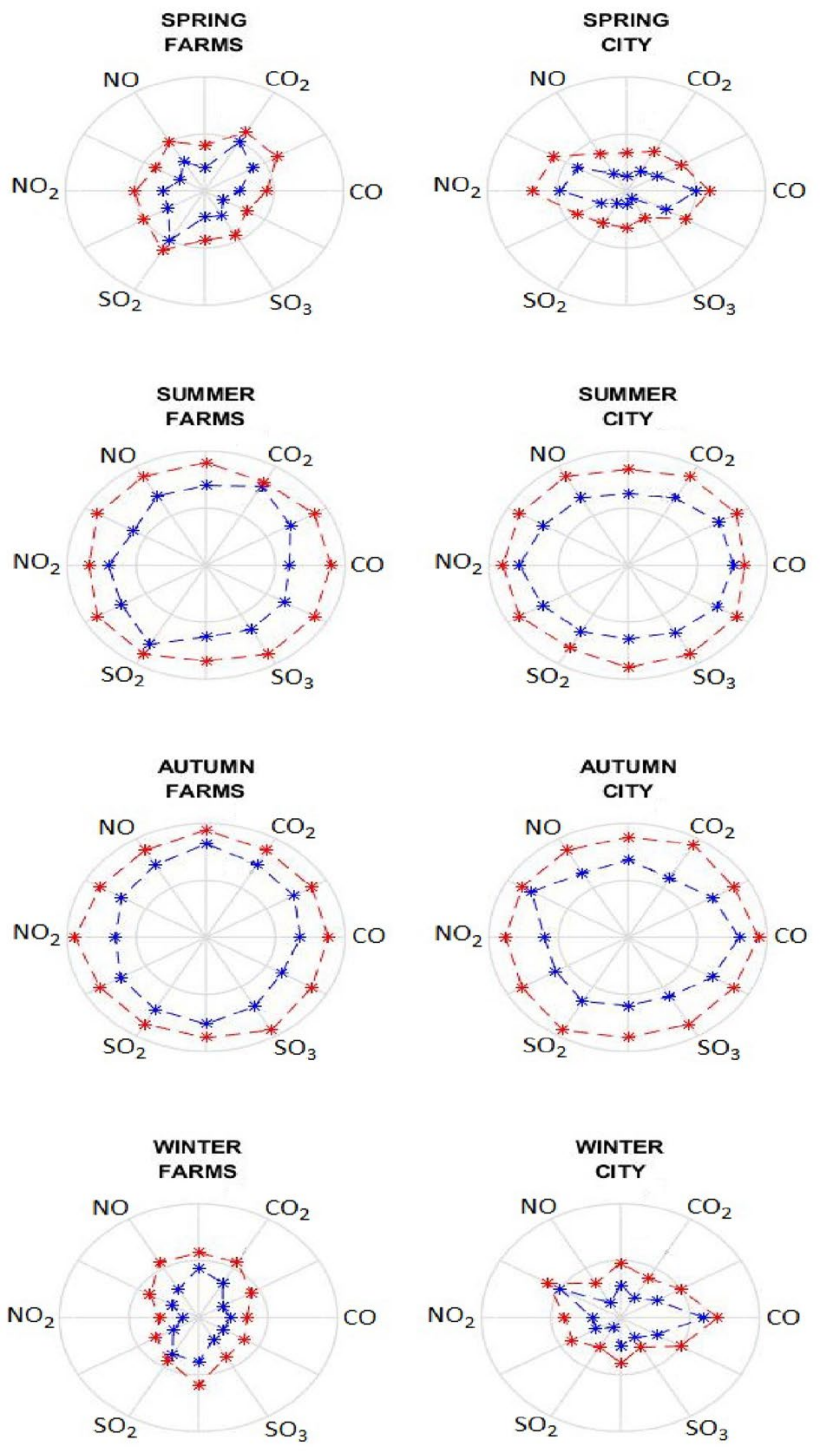
Pollutant concentrations in target areas for various seasons optimal (navy) normal (rose).

In order to verify our outcomes on optimal flare layouts most correct way is to evaluate pollutant concentrations in target areas versus normal situations in which all flares work. For an easy comparison one can report an average value for each air pollutant in residential area as well as farms. Two main air contaminants that show high amounts are $\mathrm{CO}$ plus ${\mathrm{CO}}_{2}$ that are products of complete or incomplete fuel oxidation. Furthermore, we also consider two oxides for nitrogen namely $\mathrm{NO}$ as well as ${\mathrm{NO}}_{2}$ which show high concentrations in flue gas in comparison to other ${\mathrm{NO}}_{\mathrm{x}}$ products. Yet sulfur oxides namely ${\mathrm{SO}}_{2}$ in addition to ${\mathrm{SO}}_{3}$ that can cause acidic rainfalls. Here we use polar plots to display average pollutant concentrations in target areas for normal as well as optimal layouts as shown in Fig. 5. Diagrams display average amount of each contaminant under steady state for normal conditions for which all flares are inline as well as optimal solution that artificial intelligence secures after twenty tries. In polar plots each axis illustrates a value that is relevant to one specific air pollutant. Polygons with larger area show higher mass or molar concentration while smaller ones show less perilous effects. Here we set an average value for each 4 months to report seasonal results on air pollutant concentrations in each area.

Outcomes illustrate that whenever currents are in route to our two target areas their consequences on pollutant mass fractions for optimal flare layout deviates significantly from normal flare pattern. For example, in summer that seasonal air currents are from northwest to southeast applying optimization algorithms seriously improves process performance that cause crucial reduction in pollutant emission in Kirkuk city. In another case we have autumn in which weaker air currents flow from Kirkuk refinery to farms on west. Here one can see smaller yet still important difference among pollutant mass fractions when optimal flares are inline versus normal pattern whenever all flare stacks work. Conversely during spring or winter season that we have trivial or no air flows in Kirkuk governorate air contaminants in two target areas due to sour gas flares are insignificant. In such circumstances pollutants freely arise in atmosphere to reach higher troposphere air layers with larger velocities. Surely for more accurate forecasts one can advise to concern temperature inversion for winter season which we did not include in our computations. Anyway, in slow or no air flows not only pollutant diagrams are smaller yet also difference of outcomes for two scenarios namely normal minus optimal flare layouts is unimportant. Here we schematically display two exemplar monthly optimal flare layouts for April as well as June in Kirkuk refinery as shown in Fig. 1. Main reason to choose such specific intervals is their dissimilar yet prevalent circumstances for local air currents. During a few months prior to or after April there exists low or no significant air flows in lowest troposphere in Kirkuk governorate. Conversely in one or more months no later than June or leniently July we have important local air currents in northern Iraq.

**Figure 6:**
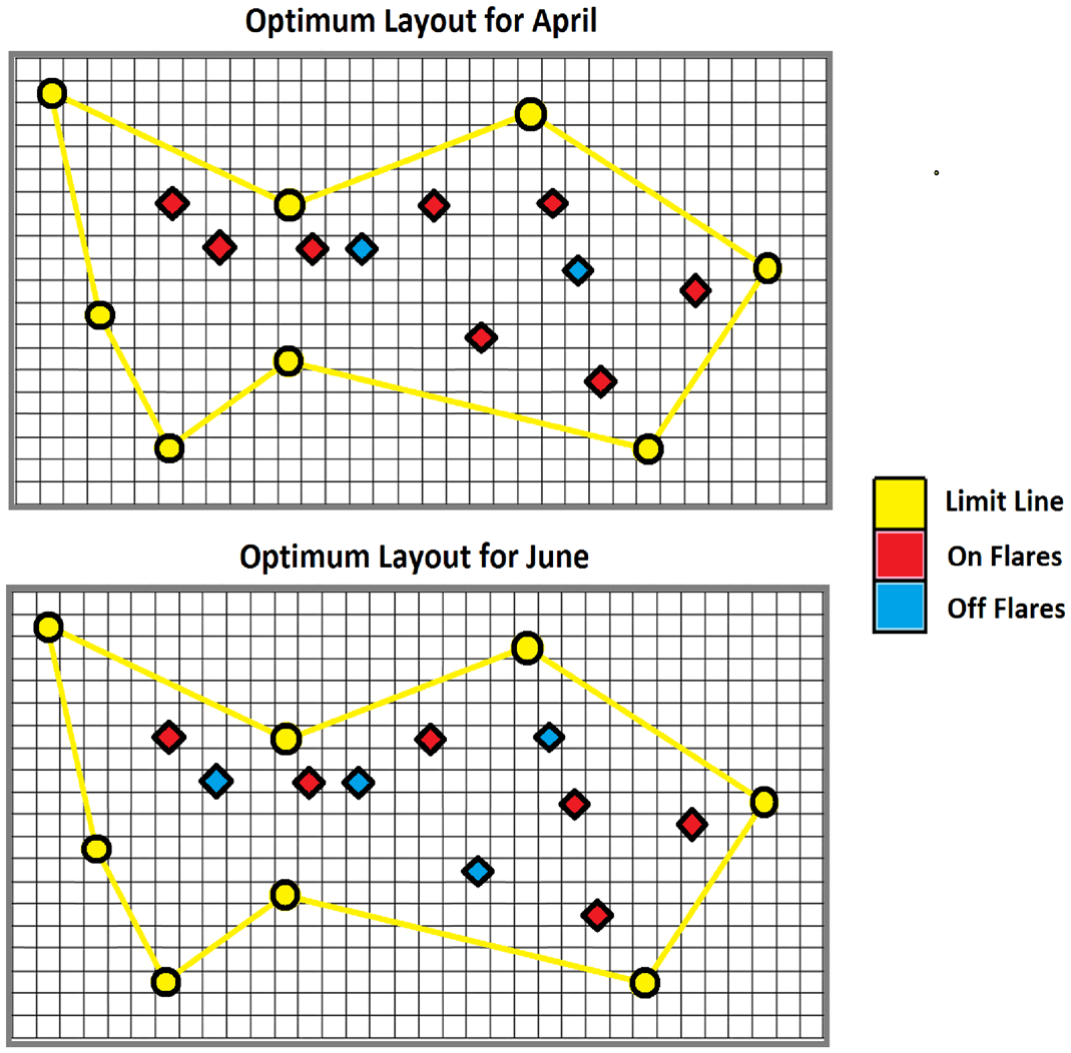
Optimal flare stacks layout in April (upper) June (lower).

Graphical results on optimum layout in April illustrate that more than 90% of existent stacks participates in sour gas oxidation. This is while in June when we have air flows direct to residential areas refinery must turn on only carefully chosen stacks up to 60% to reduce pollutant mass fractions. To discuss optimum layout for alive flares in June one can say that specific flares that sets down nearer to Kirkuk city push up pollutant gases that come from other flares. Alive stacks in optimal layout let to freely carry unequal sour gas volumetric flow rates more than minimum yet less than maximum values that flare stack can work. In addition to that each flare can have its own excess air thus specific flame temperature. One can see schematic flare layout as shown in Fig. 6. In order to speak on performance of each optimization algorithm we drew air pollutant mass fractions versus iteration for two target areas namely farms on west as well as Kirkuk city on east. By using iterations for X-axis in lieu of process runtimes one can eliminate hardware influences from process efficiency. Outcomes show that algorithms with strong global search such as Monte Carlo or particle swarm optimization are more successful to detect optimal flare layout under high air currents. Conversely artificial immune system or genetic algorithm that profit from powerful local search are useful for circumstances with low or no air currents in which selecting alive flares is important. It is noticeable that since Monte Carlo optimization algorithm has no local search capability it only gives acceptable results at high iteration. This is while particle swarm optimization profits from acceptable local search in addition to nice global search thus it can efficiently detect optimum solution for flare layouts in relatively high air currents even after insufficient tries.

**Figure 7:**
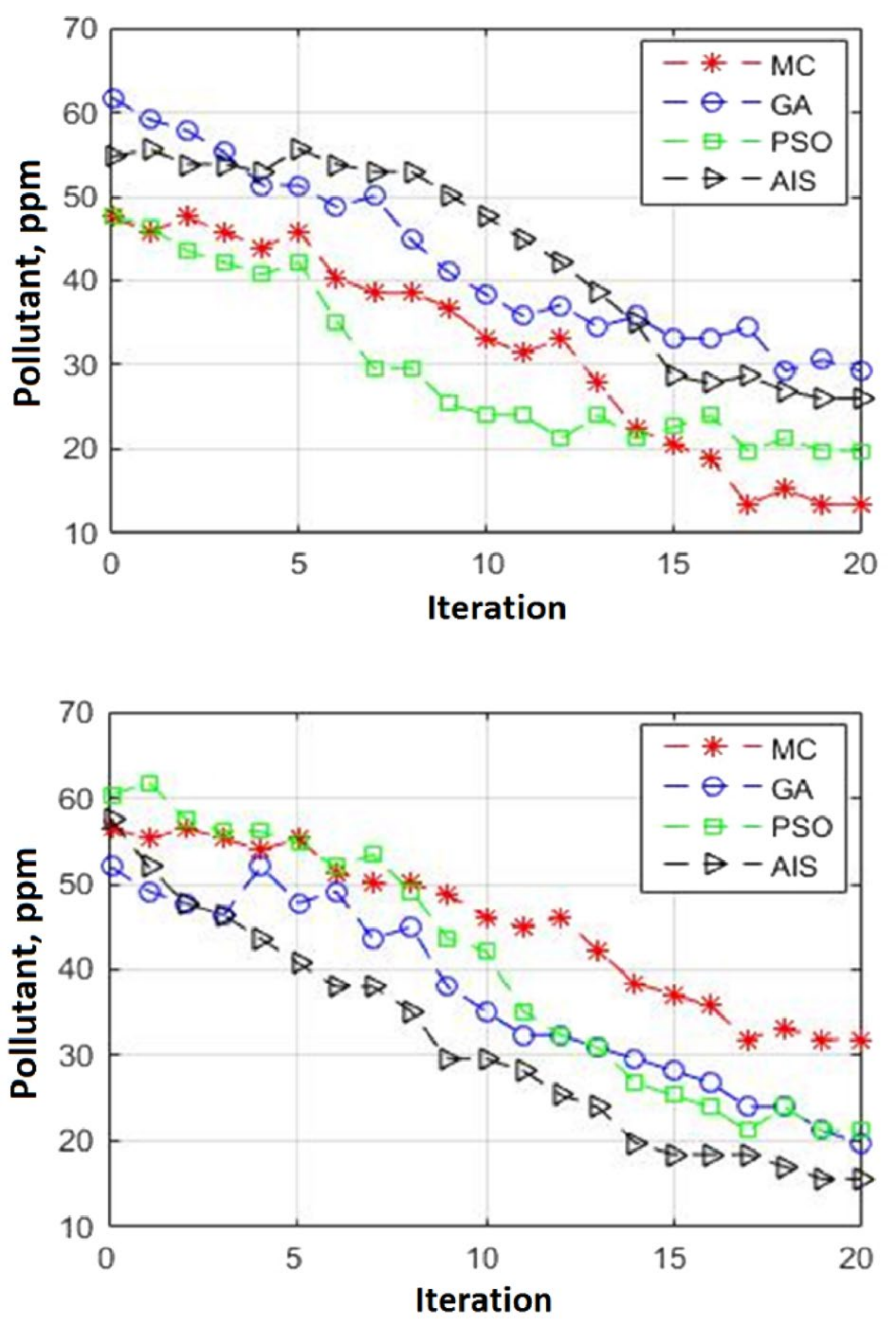
Heuristic technique performances in winter (upper) summer (lower).

At low or no air flow conditions optimization process works almost similar we mean more accurate flare stack locations attains at higher iterations except that here genetic algorithm plus artificial immune system that have strong local search impressively refine flare layout at fewer tries. In addition to that such algorithms more accurately discover optimal flare layout after achieving convergence. This is while Monte Carlo or particle swarm optimization fail to compete the two other optimization approaches from convergence viewpoint. One can look at Fig. 7 for further details. In the last step we employ neural network approach to perform sensitivity analyses on mean air flow velocity rather than direction in addition to total intake volumetric flow rate. Here artificial intelligence can rapidly forecast pollutant mass fractions in two target areas without requiring to perform computational dynamics. Here our calculations may lie under data limits that were put in to train neural network or may go out of input data limits. In first situation artificial intelligence can predict pollutant emission with high accuracy while in next state we can expect less accurate results. Since air flow velocities increase from no to low thereafter to high values pollutant mass fractions continuously increase. This is while outcomes for optimal flare layout initially are almost equal to results for normal pattern however deviates much with an increase in flow velocity moreover reduces as velocities take extremely high values. Main reason for such an action is that at no or low air currents pollutants can uprise in troposphere to reach higher layers where strong air currents carry them to farther places. Conversely when air currents are high all air contaminants will pass through target areas in circumstance where flare layout has no effect.

From flow direction point of view whenever air currents are from Kirkuk refinery to target areas namely city on east as well as farms on west pollutant mass fractions are high yet optimization process is effective. Conversely whenever seasonal air flows are not in direction of target areas low mass fractions appear thus one do not require any optimization process. In order to speak on results for sour gas volumes one can tell that at situations with high intake flow rates search for an optimal flare layout is more crucial that when refinery vents less sour gas. For further details on our analyses see Table 5.

**Table 5:** Sensitivity analysis over factors that impact optimization process.

Pollutant mass fraction	Flare layout	
	Optimum (%)	Normal (%)
Air velocity		
0 MPH	0.0001	0.0001
4 MPH	0.0003	0.0008
10 MPH	0.0007	0.0010
20 MPH	0.0013	0.0013
Air direction		
South North	0.0000	0.0000
North South	0.0003	0.0004
East West	0.0007	0.0011
West East	0.0006	0.0010
Gas flow		
$1\times {10}^{7}$ ${\mathrm{ft}}^{3}/\mathrm{D}$	0.0003	0.0004
${4\times 10}^{7}$ ${\mathrm{ft}}^{3}/\mathrm{D}$	0.0005	0.0007
${7\times 10}^{7}$ ${\mathrm{ft}}^{3}/\mathrm{D}$	0.0007	0.0011
${9\times 10}^{7}$ ${\mathrm{ft}}^{3}/\mathrm{D}$	0.0009	0.0014

## Conclusions

Several key findings from present article are as follows:

1. Either pollutant mass fractions in target areas or optimization process efficiency rely on air current velocities as well as its direction. At no or low air flows or when air velocities are extremely high optimum layout includes almost all existent flares.
2. Heuristic techniques that have stronger local search such as particle swarm or artificial immune system can improve flare layout in seasons with intermediate air currents here summer plus early months in autumn accurately at less iteration.
3. Optimization approaches which express weak local search like Monte Carlo help to detect optimal layout for flare stacks in circumstances with low or no air currents. These approaches are more appropriate in winter or spring for which we have low or no air flows in lowest troposphere layer in Kirkuk governorate.
4. Fast simulation approaches such as neural network is several times faster than full computational dynamics specially when working with large volume data whereas convergence times are almost same when working with small volume data.

## Data Availability

All data generated or analysed during this study are included in this published article.
